# Synergistic Thermal–Electrical Modulation of Broadband Terahertz Absorption via Asymmetric MoS_2_/VO_2_ Hybrid Metasurfaces

**DOI:** 10.3390/ma19143133

**Published:** 2026-07-21

**Authors:** Xiaoyue Lu, Xianbin Zhang, Shihan Zhao, Huiyu Liu

**Affiliations:** School of Optoelectronic Science and Intelligent Instruments, Xi’an University of Technology, Xi’an 710054, China

**Keywords:** terahertz metamaterial absorber, broadband absorber, MoS_2_/VO_2_ composite structure, dual-tunability mechanisms, asymmetric nested design, mode hybridization

## Abstract

**Highlights:**

- Geometric asymmetry breaks symmetry protection, inducing strong dark–bright mode hybridization for THz absorption.
- Across 1.88–3.52 THz (61% bandwidth), >96.7% absorption is achieved with 60° angular stability and ±10% tolerance.
- The thermal phase transition of VO_2_ enables reversible switching between narrowband and broadband absorption states.
- The electrical modulation of MoS_2_ carrier concentration allows for the continuous fine-tuning of the absorption spectral profile.
- Synergistic dual-tuning resolves the trade-off between bandwidth extension and dynamic device reconfigurability.

**Abstract:**

To address the challenge of simultaneously achieving broadband absorption, multi-mechanism tunability, and angular stability in terahertz multifunctional devices, this paper proposes a MoS_2_/VO_2_ composite terahertz metamaterial absorber based on an asymmetric multi-nested C-shaped structure. The device adopts a three-layer configuration consisting of a MoS_2_/VO_2_ composite plane–SiO_2_ dielectric–Au reflector layer. Unlike conventional symmetric structures, which are limited by selection rules and symmetry-protected dark modes that hinder the excitation of higher-order resonances, this design effectively breaks structural symmetry protection through geometric asymmetry. This induces strong mode hybridization between originally orthogonal dark and bright modes, enabling broadband high absorption exceeding 96.7% across the 1.88–3.52 THz frequency range (61% RBW). Notably, the device demonstrates synergistic tuning advantages: the macroscopic on/off switching of broadband absorption characteristics via the phase transition of VO_2_, combined with fine blind-spot compensation and enhancement in absorption peaks using the electrical tunability of MoS_2_. Furthermore, thanks to its sub-wavelength unit cell design, the structure maintains excellent performance stability over a wide incident angle range from 0° to 60°. This study reveals a synergistic enhancement mechanism combining the asymmetric unit cell and hybrid materials, providing a systematic physical solution for resolving the trade-off between bandwidth extension and dynamic reconfigurability.

## 1. Introduction

Terahertz (THz) waves lie between microwaves and infrared waves (0.1–10 THz). Due to their non-ionizing property, the high penetration ability of non-metallic materials, and unique fingerprint-like spectral characteristics, they have shown broad application prospects in imaging [1], next-generation communication [2], and biochemical sensing [3,4]. Meanwhile, metamaterials based on artificial structural units have been widely studied in multiple frequency ranges (including mid-infrared and terahertz bands) due to their flexible control capabilities over the amplitude, phase, and polarization of electromagnetic waves [5]. However, the electromagnetic response of natural materials in the terahertz band is relatively weak, which limits the progress of the development of efficient functional devices.

Metamaterials, as artificially designed periodic structures, can manipulate the amplitude, phase, and polarization of electromagnetic waves through sub-wavelength unit cells, exhibiting exotic properties such as a negative refractive index [6] and electromagnetic-induced transparency [7,8]. Among metamaterial-based devices, terahertz absorbers have become core components for modulators, detectors, and stealth devices due to their ability to achieve the perfect absorption of incident waves [9].

To achieve the dynamic control of terahertz absorbers, researchers have explored various methods. In recent years, in addition to traditional metal resonant units, tunable sub-wavelength resonators constructed using active materials have attracted much attention due to their design flexibility. For instance, Han and Lakhtakia proposed a thermally tunable terahertz metamaterial structure based on semiconductor open resonant rings (SRRs), which achieves resonance frequency control by adjusting semiconductor carrier concentration; Serebryannikov et al. designed a transmission-type terahertz metasurface based on VO_2_ splitting rings and grid structures, achieving polarization state switching driven by a phase transition [10,11]. These studies demonstrate that non-metallic materials can also construct sub-wavelength resonant units with dynamic control capabilities.

Among the numerous candidate materials, vanadium dioxide (VO_2_) is one of the phase-change materials that exhibits a significant change in electrical conductivity by several orders of magnitude during the transition from metal to insulator. It is an ideal candidate for achieving thermal control modulation [12,13]. Based on this property, various VO_2_-based metasurfaces have been developed for switchable and tunable terahertz absorbers [14,15]. Molybdenum disulfide (MoS_2_) is an emerging two-dimensional material, which exhibits excellent electrically tunable photonic properties and can change its carrier concentration by applying an external bias voltage [14,16,17]. In recent years, research based on these two materials has made significant progress. For instance, Liu et al. used patterned VO_2_ to achieve a thermally controllable absorber [18]; Jung et al. achieved continuous frequency shift through the ion liquid gate control of MoS_2_ [19]; Chen et al. designed a dual-mechanism modulator by combining VO_2_ with graphene [20]. Wang et al. also recently reported a broadband absorber based on a multilayer stacked structure [21].

However, there are still significant challenges in achieving the synergistic optimization of broadband high absorption, multi-mechanism tuning, and large-angle stability. Firstly, existing single-material devices are limited by the intrinsic dispersion of the materials and thus cannot achieve ultra-wideband response within a single-layer structure. Secondly, a deeper physical limitation lies in the fact that traditional designs often employ highly symmetric resonant units (such as cross-shaped and ring-shaped ones) [21,22,23,24]. This symmetry protection restricts the excitation of high-order dark modes. This symmetry bottleneck makes it difficult for multi-mode coupling to smoothly connect within a wide frequency band, thereby limiting the further expansion of bandwidth. Although dual-mechanism tuning has been reported, most of these studies merely focus on the simple superposition of material functions, lacking an exploration of the deep physical synergy between asymmetric breakdown and heterogeneous material loss. Currently, how to induce strong mode hybridization within a single-layer superstructure by breaking geometric symmetry and achieve the dynamic repair of impedance matching through different adjustment mechanisms remains a key unresolved issue.

In response to the above issues, this paper proposes a MoS_2_/VO_2_ composite terahertz absorber based on an asymmetric multi-nested C-shaped structure. Different from traditional designs, this study directly disrupts the mirror symmetry of the structure by introducing geometric asymmetry, aiming to induce strong near-field coupling between the dark mode and the bright mode. By combining the thermal phase transition of VO_2_ and the electrical control characteristics of MoS_2_, we achieved a deep synergy between structural resonance and material loss within a single-layer plane. Unlike the existing dual-tuned structures that mainly achieve frequency shift or amplitude modulation through changes in material parameters, this study further utilizes the multi-mode hybridization mechanism induced by asymmetric units to structurally construct a broadband absorption platform. On this basis, it superimposes dual-mechanism regulation to achieve a collaborative framework of macroscopic function switching + microscopic fine compensation.

The results show that this design not only achieves a relative bandwidth of 61% within the 1.88–3.52 THz frequency range but also demonstrates the complementary role of the thermal/electrical dual mechanism in impedance matching control. Through time-domain coupled mode theory (CMT) analysis, we physically revealed the unique advantages of the asymmetric unit cell in exciting multi-order resonance networks. This provides a novel design paradigm for the development of high-performance and reconfigurable terahertz integrated devices.

## 2. Materials and Methods

### 2.1. Structure Design

**Figure 1:**
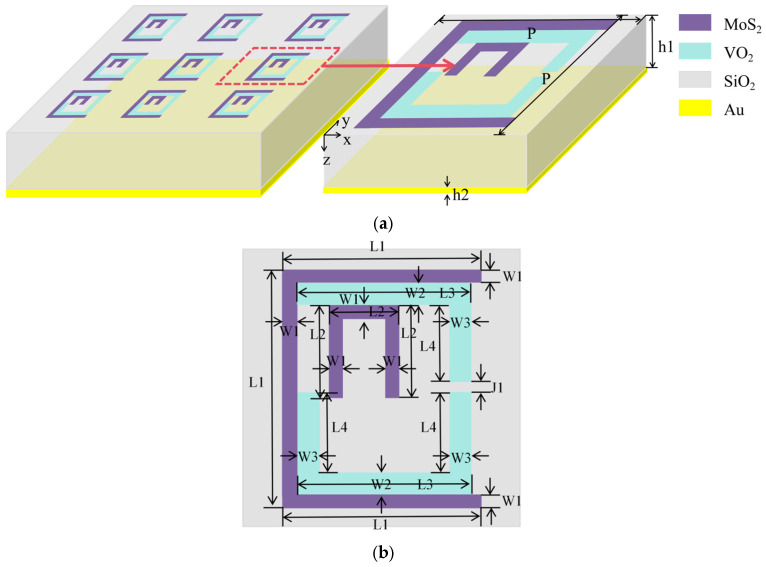
Schematic diagram of asymmetric multi-nested C-shaped MoS_2_/VO_2_ composite terahertz absorber: (**a**) three-dimensional perspective view of absorber; (**b**) top view of absorber.

This study proposes an asymmetric multi-nested terahertz absorber based on MoS_2_ and VO_2_. The absorber adopts a three-layer structure, as shown in Figure 1.

**Top Layer (Composite Metasurface):** This metasurface is composed of asymmetric multi-nested C-unit elements with a periodicity of 16 μm. The length of the outer MoS_2_ strips is 13 μm (L_1_), and the width is 1 μm (W_1_); the length of the inner nested MoS_2_ strips is 5 μm (L_2_). The lengths of the VO_2_ strips are 11 μm (L_3_) and 5.3 μm (L_4_), with widths of 1 μm (W_2_) and 1.5 μm (W_3_), respectively. The thickness of the VO_2_ film is fixed at 150 nm. All the above geometric parameters were obtained through systematic optimization to achieve broadband impedance matching within the 1.88–3.52 THz frequency range. J1 = 0.4 μm.

**Dielectric Layer (Intermediate Layer):** This layer uses loss-free SiO_2_ with thickness h1 = 13.2 μm. Its dielectric constant is set to ε_SiO2_ = 3.9. This layer functions as a Fabry–Pérot (F-P) resonator, with normalized impedance optimized by adjusting its thickness.

**Bottom Layer (Reflective Layer):** This layer uses a gold (Au) back reflection layer with thickness h2 = 1 μm (due to its thickness being far greater than the skin depth in the terahertz band, transmission is effectively suppressed (T ≈ 0)), with conductivity σ = 4 × 10^7^ S/m.

The electromagnetic response of the structure was simulated using Lumerical FDTD Solutions, which is based on the finite-difference time-domain (FDTD) method.

### 2.2. Theoretical Model

#### 2.2.1. Electromagnetic Models of Tunable Materials

In the terahertz band, the electromagnetic responses of single-layer MoS_2_ and the phase-change material VO_2_ are both dominated by intraband carrier transitions. Their complex dielectric constants $\varepsilon_{\omega }$ can be uniformly described using the Drude model [25,26]:

(1) $$\varepsilon_{\omega }=\varepsilon_{\infty }-\frac{\omega_{p}^{2}}{\omega^{2}+i\gamma \omega }$$

In this formula, $\varepsilon_{\infty }=12$ represents the dielectric constant at high frequency, ω is the angular frequency of the incident wave, $\omega_{p}$ is the plasma frequency, and $\gamma =5.75\times 10^{13}$ rad/s is the collision frequency.

For monolayer MoS_2_, its surface conductivity is defined as follows [27]:

(2) $$\sigma =\frac{ne^{2}\tau }{m^{*}\left(1-i\omega \tau \right)}$$

In this equation, *n* is the carrier concentration, e is the electron charge, *τ* = 0.17 ps denotes the intrinsic relaxation time, m* denotes the effective electron mass (m* = 0.53 m_e_), and ω represents the incident wave angular frequency.

In the simulation, monolayer MoS_2_ is modeled as an effective bulk material with an adequate thickness of *t_m_* = 0.65 nm, whose equivalent dielectric constant $\varepsilon_{m}$ can be expressed as follows [25]:

(3) $$\varepsilon_{m}=1+\frac{i\sigma }{\omega \varepsilon_{0}t_{m}}$$

where $\varepsilon_{0}$ is vacuum permittivity, and ω is angular frequency. At this point, the relationship between the plasma frequency $\omega_{p}$ and the carrier concentration *n* is given by the following:

(4) $$\omega_{p}=\sqrt{\frac{ne^{2}}{\varepsilon_{0}m^{*}}}$$

where $\omega_{p}$ denotes the plasma frequency, *n* is the carrier concentration, e is the electron charge, $\varepsilon_{0}$ is vacuum permittivity, and m* = 0.53 m_e_ denotes the effective electron mass.

In this experiment, the carrier concentration of MoS_2_ can be globally and dynamically adjusted through ion gel gate control technology (at relatively low voltages (5–10 V), the carrier concentration can be continuously regulated within the range of 0.8 × 10^14^ cm^−2^ to 1 × 10^15^ cm^−2^). This method achieves the synchronous and uniform control of all sub-wavelength units by applying gate bias voltage to the electrolyte layer covering the entire metasurface array, without the need for separate complex electrical connections. The range of carrier concentration adjustment on the surface of single-layer MoS_2_ is determined based on existing terahertz experimental studies. It has been reported in the existing literature that through ion gel gate control, the carrier concentration of single-layer MoS_2_ can continuously change within the range of 10^13^–10^15^ cm^−2^ [15,17]. Therefore, in this paper, the range of 0.8 × 10^14^ to 1 × 10^15^ cm^−2^ is selected for regulation, which is completely within the achievable range of the experiment and has practical physical feasibility.

The metal–insulator transition (MIT) of VO_2_ occurs near the critical temperature and also exhibits a Drude-type response. The high-frequency dielectric constant $\varepsilon_{\infty }=12$, and the collision frequency γ is set to 5.75 × 10^13^ rad/s [24]. The conductivity σ fluctuates dramatically during the phase transition and drives the change in $\omega_{p}$. To ensure the rigor of the results, all simulations in this paper use the heating branch data of VO_2_, setting the conductivity of the insulating state (25 °C) to σ ≈ 30 S/m and the conductivity of the fully metallic state (75 °C) to σ ≈ 9 × 10^4^ S/m.

#### 2.2.2. Absorption Mechanisms and Impedance Matching

When terahertz waves are incident perpendicular to the absorber surface, absorption is defined as follows:

(5) $$A\left(\omega \right)=1-R\left(\omega \right)-T\left(\omega \right)=1-{\left|S_{11}\left(\omega \right)\right|}^{2}-{\left|S_{21}\left(\omega \right)\right|}^{2}$$

where $R\left(\omega \right)={\left|S_{11}\left(\omega \right)\right|}^{2}$ represents reflectance, and $T\left(\omega \right)={\left|S_{21}\left(\omega \right)\right|}^{2}$ denotes transmittance [9,28].

Due to the bottom metal layer (Au) having sufficient thickness far exceeding the typical skin depth, it completely blocks transmission $T\left(\omega \right)={\left|S_{21}\left(\omega \right)\right|}^{2}=0$. Therefore, the total absorption $A\left(\omega \right)=1-R\left(\omega \right)$.

To achieve perfect absorption, the equivalent input impedance Z(ω) of the absorber must match the free-space impedance Z_0_. According to effective medium theory (EMT), the normalized equivalent impedance Z can be expressed as follows [29]:

(6) $$Z=\sqrt{\frac{(1+S_{11}{)}^{2}-S_{21}^{2}}{{\left(1-S_{11}\right)}^{2}-S_{21}^{2}}}$$

When impedance matching conditions are satisfied—that is, Z(ω) ≈ Z_0_ and Im(Z) ≈ 0—reflection is minimized, thereby achieving near-perfect absorption.

## 3. Analysis and Discussion

### 3.1. Absorption Characteristics and Physical Mechanism

#### 3.1.1. Broadband Absorption Performance

**Figure 2:**
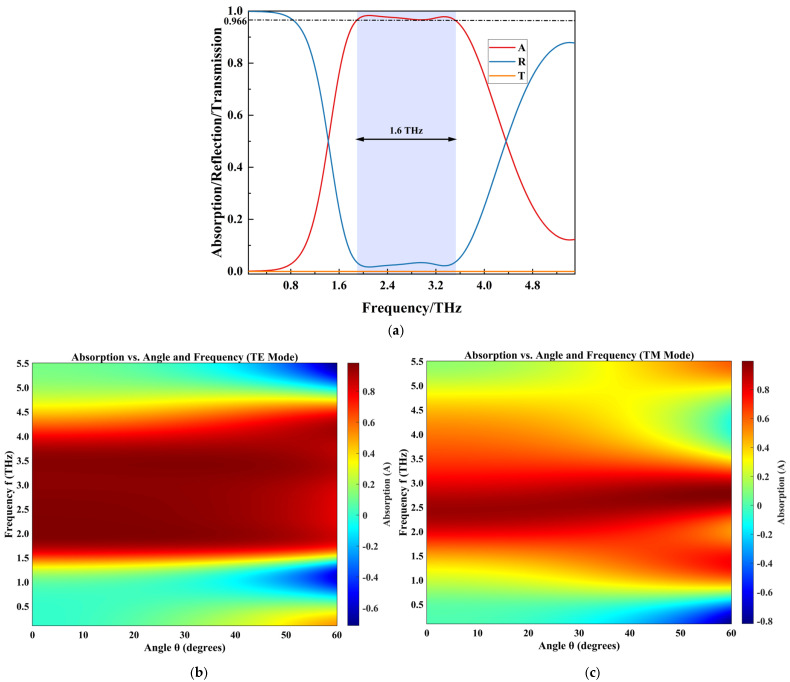
Spectral response and angular stability of absorber: (**a**) TE mode polarized spectrum; (**b**) TE mode polarized angular stability; (**c**) TM mode polarized angular stability.

When incident light is transverse electric (TE), and VO_2_ is in its metallic state (at 75 °C, with a corresponding conductivity of 9 × 10^4^ S/m), the spectral response of the designed asymmetric multilayered nested MoS_2_/VO_2_ composite absorber is as shown in Figure 2a.

The results show that this device achieves an efficient absorption of over 96.7% within a wide frequency band of 1.88–3.52 THz, with a relative bandwidth (RBA) as high as 61%, demonstrating nearly perfect absorption characteristics. Due to the thickness of the underlying gold layer being much greater than the skin depth, the transmission rate T is nearly zero throughout the frequency band, establishing the zero transmission characteristic of the device. The extremely low reflectivity (R < 1%) confirms that excellent impedance matching has been achieved between the superstructure and free space within the wide frequency band.

This broadband absorption is attributed to the multi-mode resonant coupling and mode hybridization mechanisms. This asymmetric unit cell breaks the traditional geometric symmetry, exciting the previously inaccessible high-order plasmonic modes and inducing strong near-field coupling with the fundamental modes of MoS_2_ and VO_2_. This coupling effect leads to the splitting and broadening of absorption peaks, causing multiple discrete absorption peaks to overlap in the spectrum and eventually merge to form a flat and continuous high-absorption plateau.

Figure 2b shows the variation in the absorption rate with frequency and incident angle (θ) under TE polarization conditions. When θ increases from 0° to 60°, the absorption rate within the 1.88–3.52 THz frequency band remains above 96.7%, and the central frequency does not undergo significant deviation. This excellent angle stability is mainly attributed to two factors: Firstly, the unit period of the superstructure is at the sub-wavelength scale, effectively suppressing the parasitic diffraction effect. Secondly, the localized surface plasmon resonance (LSPR) mode has a high degree of spatial localization, and its dependence on the incident light momentum is weaker compared to the propagating-type surface plasmon. Compared to traditional symmetric structures, this design has significant advantages in angle insensitivity, which is of great significance for practical applications that may encounter wide-angle incident scenarios (such as wide-angle imaging or curved surface sensing systems).

Figure 2c further illustrates the variation in the absorption rate with frequency and incident angle under TM polarization conditions. The results show that the effective absorption bandwidth (>61%) narrows from 1.88–3.52 THz under TE polarization to 2.0–3.23 THz, with a slight shift in center frequency. This polarization-related bandwidth narrowing and center frequency shift feature are closely related to the asymmetric design of the structure. Under TM polarization excitation, the electric field direction is parallel to the longer MoS_2_ and VO_2_ strips in the structure, mainly exciting the longitudinal dipole resonance mode. The non-uniform arrangement of the asymmetric nested structure in the longitudinal direction (y direction) leads to a more concentrated equivalent plasma frequency distribution in this direction, limiting the broadening of the resonance mode. In contrast, the transverse resonance mode excited by TE polarization benefits from the multi-scale nested distribution of the structure in the x direction, which can excite more complex multi-order resonance coupling and thus allows us to obtain a wider absorption bandwidth.

Although this absorber exhibits excellent angular stability within the range of 0–60°, subtle fluctuations can still be observed in the spectra at larger incident angles (such as 50–60°). From a physical perspective, these features are attributed to the angle dependence of mode coupling strength under oblique incidence. As the incident angle θ increases, the introduction of the transverse wave vector component alters the phase matching conditions between the various resonant modes within the asymmetric unit, resulting in the weak activation of previously suppressed higher-order near-field modes (Floquet modes). Due to the significant sub-wavelength characteristics of the unit cell size (at 3 THz, P ≈ λ/6), these higher-order modes mainly manifest as localized evanescent fields with extremely low radiated energy, thus only causing a slight modulation of the spectrum without disrupting the stability of the overall broadband high-absorption platform. This further demonstrates the robustness of this asymmetric unit cell in wide-angle incidence environments.

Nevertheless, under TM polarization, this structure can still maintain an absorption rate of over 96.7% in the 2.0–3.23 THz frequency band within the incident angle range of 0–60°, indicating that it still has good angle stability characteristics. This angle stability mainly stems from the sub-wavelength unit design and the spatial localization of the LSPR mode. Therefore, although bandwidth varies due to different polarization directions, angle robustness is maintained in both polarization states, indicating that the asymmetric multi-nested structure in this design has good adaptability to different polarization states.

#### 3.1.2. Impedance Matching Verification

**Figure 3:**
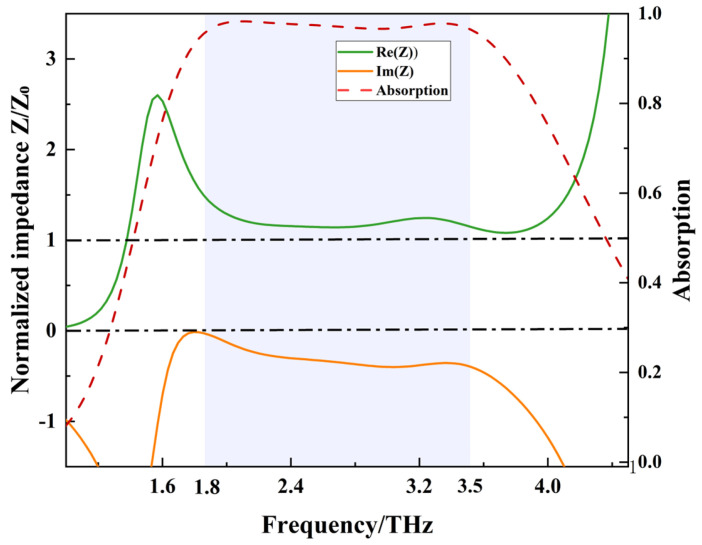
Normalized impedance and absorption spectra as functions of frequency. Re(Z): real part; Im(Z): imaginary part. The dashed lines indicate perfect matching conditions (Re(Z) = 1; Im(Z) = 0).

Figure 3 shows the frequency dependence of the real part Re(Z) and the imaginary part Im(Z) of the normalized impedance of the designed absorber and compares them with the ideal impedance matching condition (Re(Z) = 1, Im(Z) = 0). From this figure, it can be seen that within the wide frequency band of 1.88–3.52 THz, Re(Z) remains stable within the range of 1.1–1.5, and Im(Z) stays between 0 and −0.4. Although there is a certain deviation from the ideal matching values, this impedance range is sufficient to suppress the surface reflection rate to below 4% (i.e., the absorption rate >96.7%), indicating that the absorber achieves good quasi-impedance matching within this frequency band.

This result is highly consistent with the simulation data where the absorption rate is higher than 96.7%. From the perspective of equivalent medium theory, it confirms that the physical mechanism of broadband high absorption stems from the impedance approximately satisfying the matching condition over a wide frequency range. The impedance matching level achieved in this design (Re(Z) ≈ 1.1–1.5, |Im(Z)| < 0.4) is sufficient to support a broadband high absorption rate of >96.7%, which is consistent with the theoretical framework of the perfect metamaterial absorber proposed by Landy et al. [28].

#### 3.1.3. CMT Analysis

In order to deeply reveal the physical mechanism of broadband absorption, we introduced time-domain coupled mode theory (TCMT) to model the absorber. Although the full-wave FDTD simulation can provide the final electromagnetic response results, it is difficult to directly quantitatively describe the dynamic interactions between the internal resonant modes. The core role of CMT in this work is mainly reflected in the following three aspects: (1) quantitatively decomposing the flat broadband absorption platform into the contributions of several independent LSPR modes, clearly identifying the resonant frequencies and linewidths of each mode; (2) extracting the radiation loss rate ($\gamma_{r}$) and absorption loss rate ($\gamma_{a}$) and determining the system to be under-coupled, thereby revealing the matching logic of high-loss materials and structures; and (3) clarifying how the coherent interference between multiple modes enables the total absorption to exceed the limit of a single mode, thereby achieving ultra-wideband characteristics. This theoretical analysis elevates the numerical results to physical mechanisms, providing quantitative theoretical support for the design of multi-modal hybrid devices. As a classic theoretical framework for analyzing the dynamic coupling behavior of resonant systems, CMT can clearly describe the interactions, energy exchange, and contributions of multiple localized resonant modes between the local resonant modes in this structure (such as different-order LSPR modes excited by the MoS_2_ and VO_2_ bands).

For multi-mode TCMT based on the single-port reflection system, the total reflection coefficient can be expressed as follows:

(7) $$r\left(\omega \right)=-1+\sum_{j}^{N}\frac{2\gamma_{r,j}}{-i\left(\omega -\omega_{j}\right)+\left(\gamma_{r,j}+\gamma_{a,j}\right)}$$

Here, $\omega_{j}$ represents the central angular frequency of the *j*-th resonant mode, and $\gamma_{r,j}$ represents the radiative loss rate of the *j*-th resonant mode, which describes the rate at which energy radiates outward from the resonant mode and determines the brightness of the mode. $\gamma_{a,j}$ represents the absorptive loss rate of the *j*-th resonant mode, which describes the rate at which energy is dissipated within the material (mainly due to the ohmic loss of VO_2_ and the intraband transition loss of MoS_2_). Since the bottom metal layer completely blocks transmission (T ≈ 0), the absorption rate can be directly obtained as A(ω) = 1 − |r(ω)|^2^. When $\gamma_{r}=\gamma_{a}$, the system reaches the critical coupling state, and at this time, single-mode absorption is maximized.

**Figure 4:**
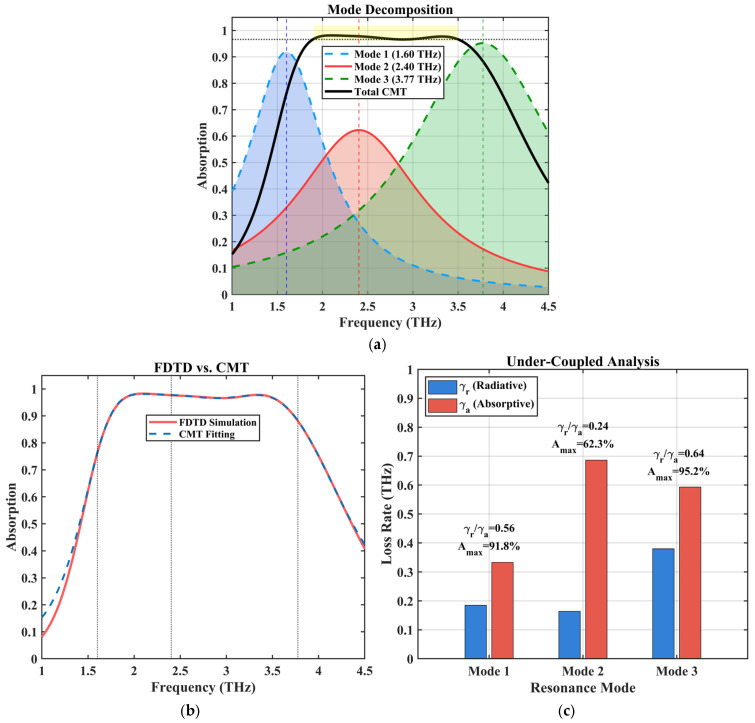
Coupled mode theory (CMT) analysis: (**a**) mode decomposition; (**b**) FDTD vs. CMT (R^2^ = 0.9997); (**c**) under-coupled analysis.

Figure 4a presents the Lorenz-type absorption contribution curves of three independent resonance modes and their superimposed results. The peak heights of each component curve represent the theoretical maximum absorption rate (A_max_) of the corresponding mode when operating independently. Unlike the traditional understanding, these three modes do not directly correspond to the peak positions of the absorption spectrum but achieve broadband coverage through a boundary–center–boundary distribution strategy: Mode 1 (blue, 1.60 THz) and Mode 3 (green, 3.77 THz) are respectively located at the low-frequency and high-frequency boundary regions of the absorption band, and their Lorenz tails extend into the band; Mode 2 (red, 2.40 THz) is located at the band center, filling the core area with its wide linewidth. Despite being limited by the deep under-coupling state, the independent absorption peak height of the central mode (Mode 2) is only 62.3%, but the three modes have significant spectral overlap within the range of 1.88–3.52 THz (the yellow marked area in the figure). This spectral overlap causes the complex reflection coefficients of each mode to undergo destructive interference, effectively suppressing overall reflectivity, enabling the total absorption spectrum (black solid line) to exceed the absorption limit of a single mode and remain above the 96.7% threshold line (black dashed line) throughout the core frequency range.

It is particularly important to note that the total absorption rate A_total(ω)_ is not simply the algebraic sum of the individual mode absorption rates A_j(ω)_ but rather results from the coherent interference effect among the reflection coefficients of multiple resonant modes. To clearly illustrate this mechanism, let us take a two-mode system as an example. The total reflection coefficient is $r_{total}=r_{1}+r_{2}$, and the corresponding absorption rate is as follows:

(8) Atotal=1−rtotal=1−r1+r22=A1+A2−2Rer1·r2¯

The cross term 2Rer1·r2¯ plays a decisive role in spectral overlap and absorption enhancement. Here, *r_j_* denotes the complex reflection component associated with the *j*-th resonant mode.

When the resonant wings of different modes overlap and interfere destructively in the frequency domain, the total reflectivity will be significantly lower than the contribution of a single mode, thereby enabling the total absorption rate Atotal to exceed the absorption limit of a single mode (such as 62.3% for Mode 2), achieving flat and high-efficiency absorption within the 1.88–3.52 THz range. This mechanism of phase compensation between modes is precisely the physical essence of the asymmetric nested structure achieving a flat broadband response.

Figure 4b presents a comparison between the FDTD full-wave simulation absorption spectrum (red solid line) and the fitting result of the three-mode CMT theoretical model (blue dashed line). It can be observed that the theoretical curve and the simulation data are highly consistent within the frequency range of 1.5–4.0 THz (the goodness of fit R^2^ = 0.9997), verifying that the CMT model can accurately reproduce the electromagnetic response of the full-wave simulation and confirming the correctness of the multi-mode coupling mechanism.

The key parameters extracted by the CMT model fitting are summarized in Table 1. The analysis shows that all three resonant modes are in an under-coupled state ($\gamma_{r}$ < $\gamma_{a}$), meaning that the absorption loss dominates the energy dissipation process. This characteristic is closely related to the material properties of the composite structure, the strong ohmic loss of the metallic state VO_2_ (σ = 9 × 10^4^ S/m) and the intraband transition loss of MoS_2_ jointly contribute to a significant absorption loss rate.

Figure 4c presents the coupling states of each mode in a bar chart format. For each mode, the blue bar represents the radiation loss rate $\gamma_{r}$, and the red bar represents the absorption loss rate $\gamma_{a}$. The results show that the radiation loss rate (blue) of all modes is less than the absorption loss rate (red), confirming the under-coupling feature. It is notable that the coupling ratio of Mode 2, κ2 = 0.24, is significantly lower than that of Mode 1 (κ1 = 0.56) and Mode 3 (κ3 = 0.64), demonstrating a deeper under-coupling feature. This is mainly due to the fact that Mode 2 is mainly localized in the inner strips of the asymmetric unit cell (such as V3, V4) and is affected by the electromagnetic shielding effect of the outer structure, resulting in the suppression of its energy back radiation to free space (the radiation loss rate $\gamma_{r}$ is smaller). At the same time, VO_2_ in the metallic state exhibits extremely strong intrinsic ohmic loss in this frequency band, further increasing the absorption loss rate $\gamma_{a}$ and bringing the system into a deep under-coupling state. However, it is precisely this high-loss characteristic induced jointly by the material properties and the structure layout that provides sufficient energy traps for energy dissipation over a wide bandwidth, ensuring the smoothness and continuity of spectral transitions between different resonant modes.

According to CMT, the maximum absorption rate of the single mode is as follows:

(9) $$A_{\max,j}=\frac{4\gamma_{r,j}\gamma_{a,j}}{{\left(\gamma_{r,j}+\gamma_{a,j}\right)}^{2}}$$

The single-mode theoretical maximum absorption rates of the three modes were calculated to be 91.9% (Mode 1), 62.3% (Mode 2), and 95.3% (Mode 3). It is worth noting that although the single-mode maximum absorption rate of Mode 2 was only 62.3%, its extremely high absorption loss rate ($\gamma_{a}$ = 0.686 THz) ensured that the electromagnetic energy coupled into the structure was rapidly dissipated, avoiding energy re-radiation.

To reveal the influence of the material phase transition on modal coupling characteristics, we performed the same CMT fitting on the insulating state of VO_2_ (at 25 °C) under the condition of a fixed high carrier concentration of MoS_2_ (see Table 1). The comparison results show that the absorption loss rate $\gamma_{a}$ of Mode 2 significantly decreased from 0.686 THz in the metallic state to 0.124 THz, while the quality factor Q increased from 1.41 to 6.17, indicating that the system changed from a broadband under-coupled state to a narrowband localized resonance-dominated state. Additionally, the coupling ratio κ increased from 0.24 to 0.78, suggesting a significant change in the balance relationship between the radiation and absorption channels. Due to the reduction in VO_2_ electrical conductivity by three orders of magnitude during the phase transition (from 9 × 10^4^ S/m to 30 S/m), the material’s dissipation channels significantly weakened, and the multi-mode cooperative coupling condition was disrupted, resulting in the collapse of the broadband absorption platform into a narrowband resonant mode.

The actual device achieved a broadband high absorption of over 96.7% within the range of 1.88–3.52 THz. This beyond single-mode limit feature can be attributed to the following physical mechanisms:

(1) Multi-mode spectral superposition effect: The Lorentzian lines of the three resonant modes overlap in the frequency domain, forming a quasi-continuous absorption platform. In the overlapping area, the absorption contributions of each mode are coherently superimposed, effectively compensating for the insufficient absorption caused by single-mode under-coupling.

(2) Boundary mode defined bandwidth: As for Mode 1 (1.60 THz) and Mode 3 (3.77 THz), although they are located outside the high absorption platform, their Lorentzian tails extend to the core region of 1.88–3.52 THz, respectively defining the low-frequency and high-frequency boundaries of the absorption band.

(3) Central mode support strength: Mode 2 (2.40 THz) is located at the center of the bandwidth, and although it has the deepest under-coupling degree, its wide linewidth and high absorption loss rate ensure efficient energy dissipation in the core frequency band.

(4) Material cooperative loss: Based on the analysis in Section 3.2.1, the ohmic loss of the VO_2_ metal state and the band–intraband transition loss of MoS_2_ cooperate in the composite structure, enhancing overall absorption efficiency.

### 3.2. Structural Parameter Optimization and Mechanism Analysis

Structural parameters are one of the key factors influencing the performance of the absorber. By analyzing parameters such as the geometric dimensions and pattern design of the absorber, we can precisely adjust its performance.

#### 3.2.1. Material Synergy Analysis

**Figure 5:**
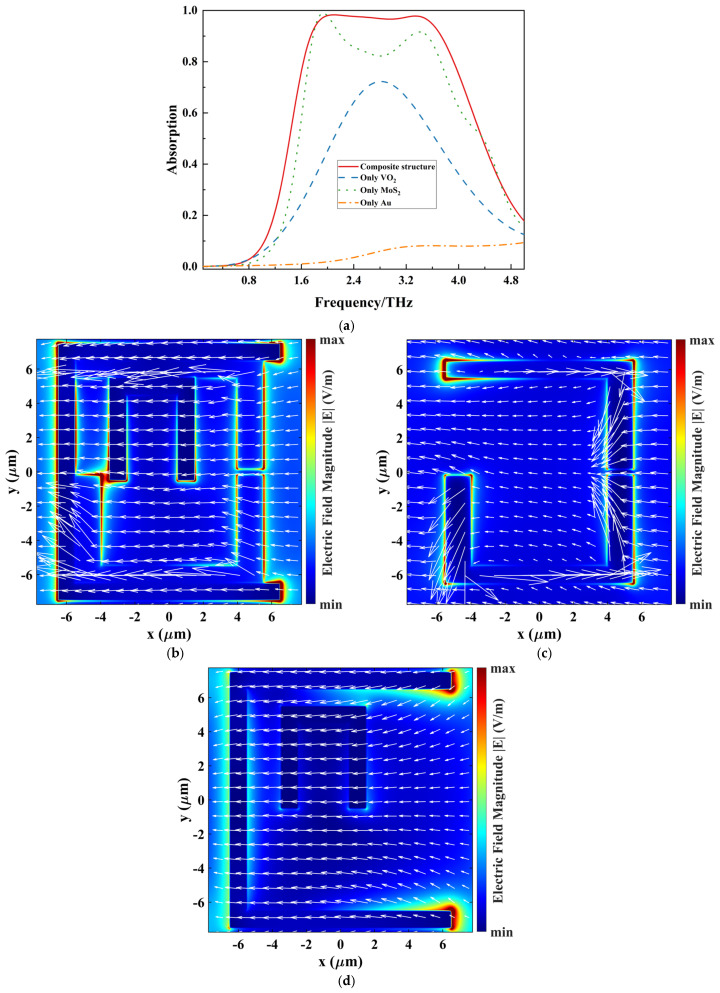

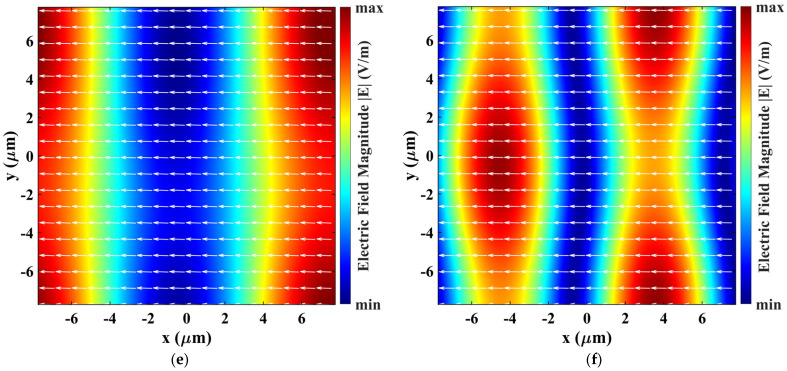
Comparison of absorption spectra for different materials and corresponding electric field/current distributions: (**a**) Comparison of absorption spectra for absorbers with different material compositions. (**b**) Electric field and current density distribution of composite structure. (**c**) Electric field and current density distribution of structure containing only VO_2_. (**d**) Electric field and current density distribution of structure containing only MoS_2_. (**e**) Electric field and current density distribution of dielectric layer. (**f**) Electric field and current density distribution of gold layer. (VO_2_: metallic state; MoS_2_: *n* = 1 × 10^15^ cm^−2^).

In order to study the synergistic effect of MoS_2_ and VO_2_ in the composite structure, Figure 5a compares the absorption spectra of the absorbers under four different material configurations.

Only the absorption spectrum of the MoS_2_ structure presents a double-peak broadband characteristic, but there is a significant absorption dip (i.e., M-type distribution) within the frequency band between the two peaks, failing to achieve a flat broadband response. Only the VO_2_ structure shows a single peak at around 2.7 THz, but due to poor impedance matching, the peak absorption rate is only 72.2%. The MoS_2_/VO_2_ composite structure exhibits significant spectral overlapping. The composite structure not only integrates the high absorption intensity of MoS_2_ and the broadband characteristics of VO_2_ but also excites new hybrid modes through interlayer and intralayer electromagnetic coupling, filling the absorption valley between the two independent resonance peaks, thereby achieving perfect broadband absorption from 1.88 to 3.52 THz.

To further quantify the absorption sources and exclude the contribution from the Au back reflector, we performed a control simulation for a reference structure consisting only of the SiO_2_ dielectric spacer and the Au reflective layer (i.e., without the top metasurface). The simulation results showed that the maximum absorption rate of the bare substrate composed of a 1 μm gold reflective layer and a 13.2 μm SiO_2_ dielectric layer throughout the entire research frequency band was only 0.1% (as shown by the orange dotted line in Figure 5a). This extremely low value clearly confirmed that the intrinsic ohmic loss of the underlying reflector has almost no contribution to the overall performance of the device, thereby strongly proving that the nearly perfect broadband absorption achieved is entirely due to the resonant dissipation caused by the MoS_2_/VO_2_ hybrid asymmetric unit.

To more systematically compare the effects of different material states on absorption characteristics, the absorption spectra of four real material state combinations, including the VO_2_ insulating state and the metallic state, are uniformly presented in Figure 9c and discussed in detail in Section 3.3. This supplementary result further verified the decisive role of the VO_2_ phase transition in the formation of broadband absorption.

Figure 5b–f further reveal the microscopic mechanism through the distribution of the electric field and current. The field distribution map visually demonstrates the degree of localization of electromagnetic energy in space, and its resonant strength has a direct physical correspondence with the peak position in the absorption spectrum. In the composite structure (Figure 5b), the electric field is not only localized at the edges of the structure but also forms a high-intensity gap plasma between the MoS_2_ and VO_2_ strips. The current distribution shows that a continuous displacement current loop is formed between MoS_2_ and VO_2_, directly confirming the strong electromagnetic coupling effect. The field distribution of the control structure further confirms the possible advantages of the composite design. Figure 5c (only VO_2_ structure) shows that the electric field is mainly localized at the edges of the VO_2_ strips, but since all the strips are of the same homogeneous material, the electromagnetic coupling between different strips is limited by the same dielectric response, with a limited degree of mode hybridization, and the absorption spectrum shows a single broad peak with a peak absorption rate of only 72.2%. Figure 5d (only MoS_2_ structure) presents an M-shaped double-peak distribution, with the electric field concentrated at the ends of different-length strips. The low-frequency peak (~1.9 THz) has a higher amplitude than the high-frequency peak (~3.3 THz), and there is an absorption depression between the two peaks. Each dipole mode is relatively independent in the frequency domain and fails to form a flat broadband response.

In contrast, the heteromaterial interface between MoS_2_ and VO_2_ in the composite structure (Figure 5b) supports stronger near-field coupling, with a higher field strength and stronger localization. Additionally, the typical feature shown in Figure 5e of a strong electric field distribution within the dielectric layer confirms the existence of the Fabry–Pérot (F-P) cavity effect, and the effective energy limitation further enhances absorption. Figure 5f shows that the underlying gold layer acts as a perfect reflector, further suppressing transmission.

MoS_2_ and VO_2_ have different plasma frequencies and damping characteristics in the terahertz band. In the asymmetric nesting structure, the local surface plasmon modes of the two materials strongly couple, forming a hybrid mode, and are manifested as the merging and broadening of absorption peaks on the spectrum. The single-material structure has a narrow impedance matching frequency band due to fixed electromagnetic parameters, while the composite structure can flexibly adjust the overall equivalent electromagnetic parameters through the cooperative effect of the materials, thus meeting the impedance matching condition (Z(ω) ≈ Z_0_) over a wider frequency range. At the same time, the intraband transition loss of MoS_2_ and the ohmic loss of VO_2_ in the composite structure jointly act, significantly enhancing the dissipation efficiency of electromagnetic energy.

#### 3.2.2. Pattern Evolution and Resonance Mode Analysis

**Figure 6:**
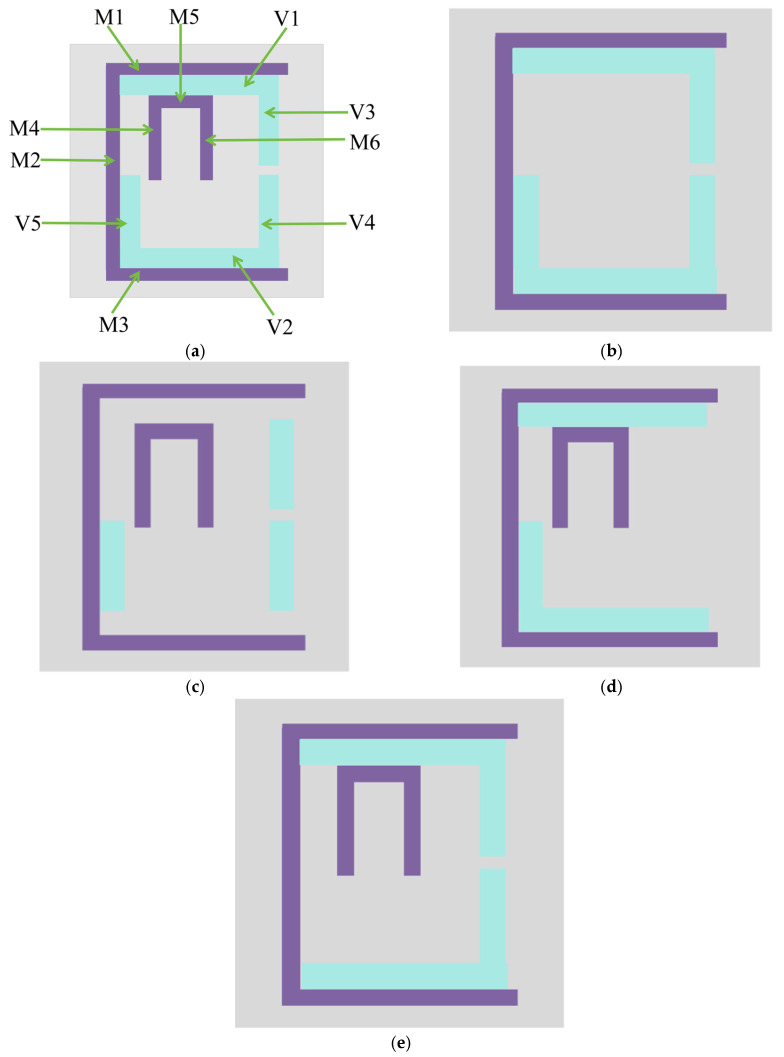

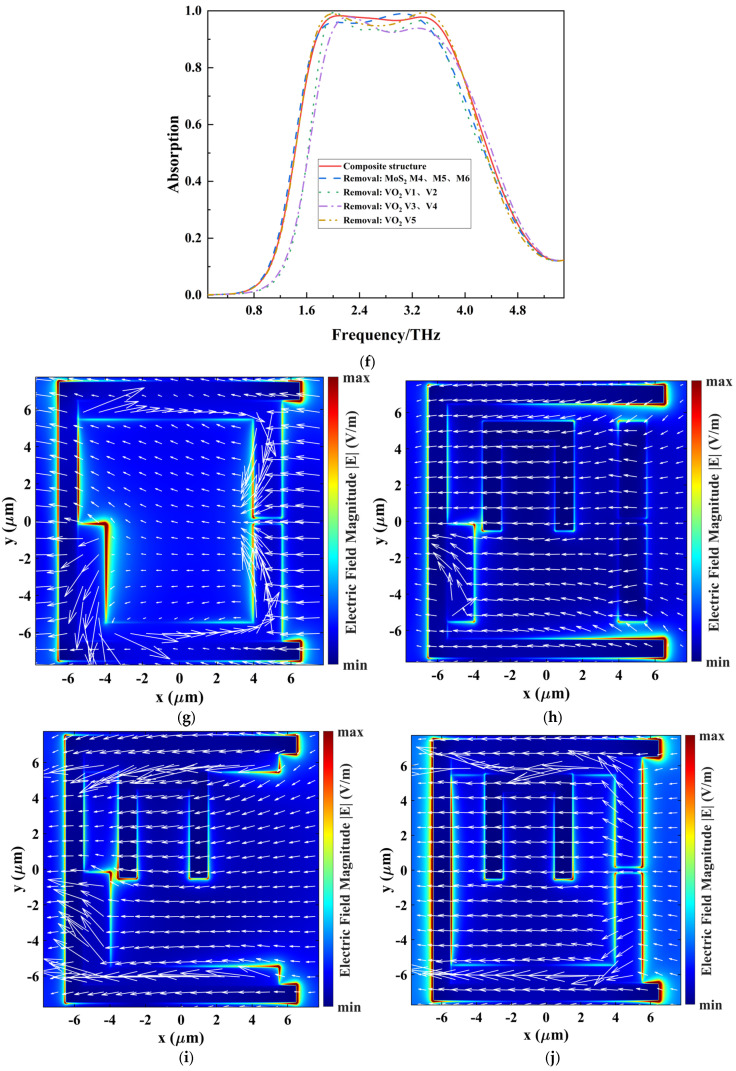
Structural evolution analysis: Comparison of absorption spectra and near-field distributions under different pattern combinations. (**a**) MoS_2_/VO_2_ forming asymmetric multi-nested C-shaped array. (**b**) Removal of MoS_2_: M4, M5, and M6 strips. (**c**) Removal of VO_2_: V1 and V2 strips. (**d**) Removal of VO_2_: V3 and V4 structures. (**e**) Removal of VO_2_: V5 structure. (**f**) Absorption spectra of different patterned absorbers. (**g**) Current and electric field distribution for removal of MoS_2_: M4, M5, and M6 strips. (**h**) Current and electric field distribution for removal of VO_2_: V1 and V2 strips. (**i**) Current and electric field distribution for removal of VO_2_: V3 and V4 structures. (**j**) Current and electric field distribution for removal of VO_2_: V5 structure. (VO_2_: metallic state; MoS_2_: *n* = 1 × 10^15^ cm^−2^).

To verify the possible advantages of this asymmetric design and to clarify the contribution of each strip to the absorption spectrum, under the condition of keeping other parameters of the structure unchanged, a removal analysis was conducted on specific parts of the structure (as shown in Figure 6). The electric field and current distributions in Figure 6g–j correspond to the field distributions of each structure at their characteristic absorption peak frequencies, as shown in Figure 6f. The geometric configuration is exactly the same as the geometric topology in Figure 6b–e, enabling us to visually compare and analyze the contributions of each unit to the resonant paths in specific frequency bands.

Figure 6f presents the absorption spectra of five structures: Regarding the removal of MoS_2_ (M4–M6), absorption in the low-frequency range (<2.8 THz) significantly decreases, while absorption in the mid-frequency range increases. From the electric field distribution in Figure 6g, it can be seen that the M4–M6 bands mainly carry the dipole resonances of the long wavelength range, and their removal cuts off the low-frequency coupling path. It can be observed in this figure that the low-frequency electric field distribution originally concentrated around these bands disappears. This not only explains the decrease in low-frequency absorption in the spectrum but also demonstrates the contribution of specific geometric units to the absorption in specific frequency bands.

The removal of VO_2_ (V1, V2) leads to a drop in the absorption rate within the 1.8–2.5 THz range and disrupts overall impedance matching, indicating that the outer VO_2_ structure plays a dominant role in low-frequency resonances and impedance regulation. Figure 6h shows that after removing V1 and V2, the overall electric field intensity of the structure significantly weakens, especially in the low-frequency region, where the electric field almost disappears.

Absorption depressions occur in the high-frequency range (2.5–3.5 THz) when VO_2_ (V3, V4) is removed, indicating that the intermediate layer bands are crucial for connecting high- and low-frequency modes and achieving spectral flattening. Figure 6i shows that the absence of V3 and V4 interrupts the electric field coupling paths in the mid–high-frequency region, resulting in a significant decrease in resonance intensity.

The removal of VO_2_ strip V5 has a relatively small impact on the absorption spectrum, but a slight decrease in the absorption rate can still be observed in the 2.2–3.2 THz frequency range, indicating that V5 plays an auxiliary role in smoothing absorption peaks and enhancing mode coupling. Figure 6j shows that the removal of V5 has a limited effect on the overall electric field distribution, with only a local weak reduction in electric field in specific frequency bands, consistent with the slight decrease in the absorption rate in the absorption spectrum. By comparing the field distribution of each structural unit in the ‘resonance network’ with the complete structure shown in Figure 5b, we can clearly understand the topological role of each structural unit: the outer strips determine the boundary frequency, and the inner strips achieve spectral filling.

It should be noted that although the differences between the various structural combinations in Figure 6f are relatively small in certain frequency bands, this result does not imply a deviation in the working mechanism. The purpose of this analysis is to identify the relative contribution of each component unit to the resonance in different frequency bands, rather than causing a complete reconstruction of the macroscopic absorption mode. Due to the synergistic superposition effect of multiple resonance modes within the broadband platform, removing a single structural unit will only weaken the local coupling path but will not completely destroy the overall absorption framework. Therefore, the differences shown in Figure 6 reflect the collaborative regulation mechanism of the multi-mode network, rather than a deviation in the working mechanism.

The above feature can be confirmed by the electromagnetic field distribution (Figure 6g–j). This series of analyses indicates that the asymmetric nested C-shaped structure essentially constructs a multi-scale resonance network, with each part of the strips responding to different frequency bands and forming a cooperative effect through near-field coupling. Removing any part will disrupt some links of the network, not only weakening the corresponding local resonance but also affecting the overall impedance characteristics through coupling effects, resulting in deviation from the matching condition (Z(ω) ≈ Z_0_) at specific frequencies, increased reflection, and decreased absorption. Therefore, the integrity of the structure is the basis for achieving broadband perfect absorption.

#### 3.2.3. Dielectric Layer Thickness Control

**Figure 7:**
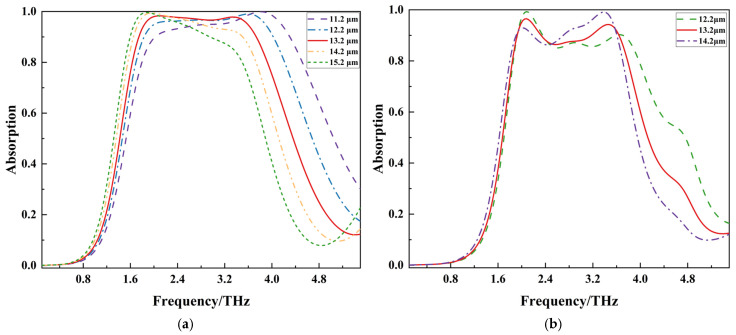
Influence of dielectric layer thickness h1 on absorption characteristics under different material states. (**a**) VO_2_ metallic state (75 °C) with MoS_2_ carrier concentration *n* = 1 × 10^15^ cm^−2^; (**b**) VO_2_ insulating state (25 °C) with MoS_2_ carrier concentration *n* = 1 × 10^15^ cm^−2^.

Figure 7 shows the influence of the thickness of the dielectric layer h1 on absorption performance. We systematically compared the thickness-dependent characteristics of VO_2_ in the metallic state (Figure 7a) and the insulating state (Figure 7b).

In the metallic state, as shown in Figure 7a, when h1 = 13.2 μm, the absorber achieved the maximum bandwidth while achieving an absorption rate >96.7%, and the absorption platform was the flattest. Therefore, it was defined as the optimal thickness. At this time, the double peaks of the absorption spectrum merged well, demonstrating the best broadband absorption characteristics. When the dielectric layer thickness was reduced to h1 = 11.2 μm, the absorption spectrum moved towards high frequency, and low-frequency absorption significantly weakened, especially in the <3.5 THz frequency band, where the absorption rate decreased significantly. When the dielectric layer thickness was increased to h1 = 15.2 μm, the absorption spectrum moved towards low frequency, high-frequency absorption weakened, and the absorption bandwidth narrowed. In the insulating state (VO_2_ insulator + high-concentration MoS_2_) shown in Figure 7b, a clear thickness modulation effect can also be observed. For instance, when h1 increases from 12.2 μm to 14.2 μm, the position of the main absorption peak undergoes a significant red shift. When the thickness shifts from 13.2 μm to 12.2 μm or 14.2 μm, the lowest absorption rate within the target frequency band drops by 85–86%, although the peak absorption remains above 95%, but it lacks broadband absorption characteristics. Although the overall absorption level in the insulating state is lower than that in the metallic state, the optimal thickness still lies around 13.2 μm.

This feature can be explained by the Fabry–Pérot resonance condition. Terahertz waves undergo multiple reflections between the upper structure and the bottom gold reflective layer, forming a standing wave. The thickness of the dielectric layer determines the phase difference between the reflected waves. The optimal thickness corresponds to the phase-matching condition, under which the reflected waves and the incident waves are coherently superimposed to enhance the local electric field. Additionally, the thickness of the dielectric layer directly affects the normalized equivalent impedance by changing the structural equivalent optical length. The optimal thickness makes Z(ω)/Z_0_ the closest to 1 within the wide frequency band.

At the same time, changes in the dielectric layer thickness will alter the coupling distance between the upper MoS_2_/VO_2_ structure and the bottom gold layer, thereby affecting the coupling strength between the high- and low-frequency resonance modes. At the optimal thickness, the optical length of the resonator satisfies the phase-matching condition within the wide frequency band, causing the reflected wave on the upper surface to undergo constructive interference with the wave reflected from the gold layer and emerging again, significantly enhancing the local electric field at the top structure, thus achieving the optimal broadband absorption performance. When the thickness deviates, this matching condition is disrupted, and the absorption peak shifts accordingly, with the bandwidth narrowing.

#### 3.2.4. Fabrication Tolerance Analysis

**Figure 8:**
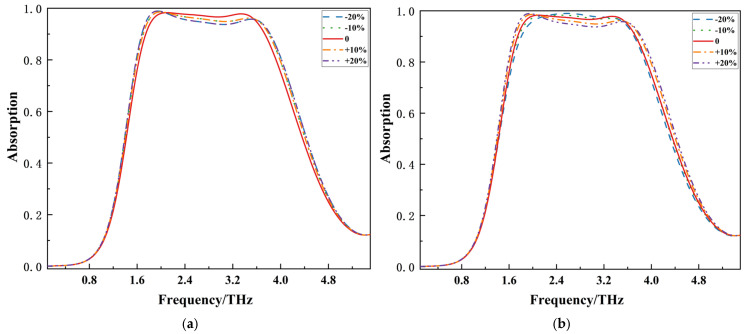
Fabrication tolerance analysis of key geometric parameters. (**a**) Influence of gap parameter J1 variation (±10%, ±20%). (**b**) Influence of strip width W1 variation (±10%, ±20%).

During the actual production process, the precision limitations of the processing equipment may cause the structural parameters to deviate from the designed values. To evaluate the sensitivity of the device to processing errors, this section analyzes the impact of the key clearance parameter J_1_ changing within the range of ±10% and ±20% on absorption performance. Figure 8a shows the change in the absorption spectrum when J1 varies from 0.32 μm (−20%) to 0.48 μm (+20%). From this figure, it can be seen that within the tolerance range of ±10% to ±20% (J1 = 0.36–0.44 μm), the absorber maintains an absorption rate of above 95.9% in the frequency band of 1.88–3.52 THz, and the shape of the absorption spectrum remains basically unchanged. At the high-frequency end (3–3.8 THz), the absorption rate slightly decreases (<1.5%), with only a slight frequency shift (<0.05 THz).

The above analysis mainly focuses on the key gap parameter J1, whose physical function is to control the near-field coupling strength between the MoS_2_ and VO_2_ strips. However, according to the structural removal analysis in Figure 6f, the MoS_2_ strips themselves also play an important role in the formation of low-frequency and wideband characteristics. Therefore, we further conducted a tolerance analysis on the narrowest conductive strip width W_1_ to evaluate the impact of processing errors on device performance.

Figure 8b shows the absorption spectra when W1 varies by ±10% and ±20%. The results indicate that the minimum absorption within 1.88–3.52 THz remains higher than 95.0% for a ±10% deviation. For a larger ±20% deviation, the minimum absorption remains above approximately 93.7%, mainly manifested as a noticeable reduction near the high-frequency edge together with a slight spectral shift.

This robustness can be attributed to the multi-mode nature of the broadband response. The broadband absorption plateau is formed by the spectral overlap of several localized plasmonic resonances with relatively low quality factors (high losses), whose broad linewidths make them less sensitive to geometric perturbations than high-Q narrowband resonances. When W1 is varied, individual resonant features may shift; however, due to the asymmetric nested geometry and inter-mode coupling, neighboring modes can provide partial compensation, thereby mitigating the overall degradation of the broadband absorption platform. These results demonstrate that the proposed design exhibits good tolerance not only to the gap parameter J1 but also to variations in the strip width W1.

### 3.3. Dual-Mechanism Dynamic Tuning: Thermal Switching and Electrical Fine-Tuning

**Figure 9:**
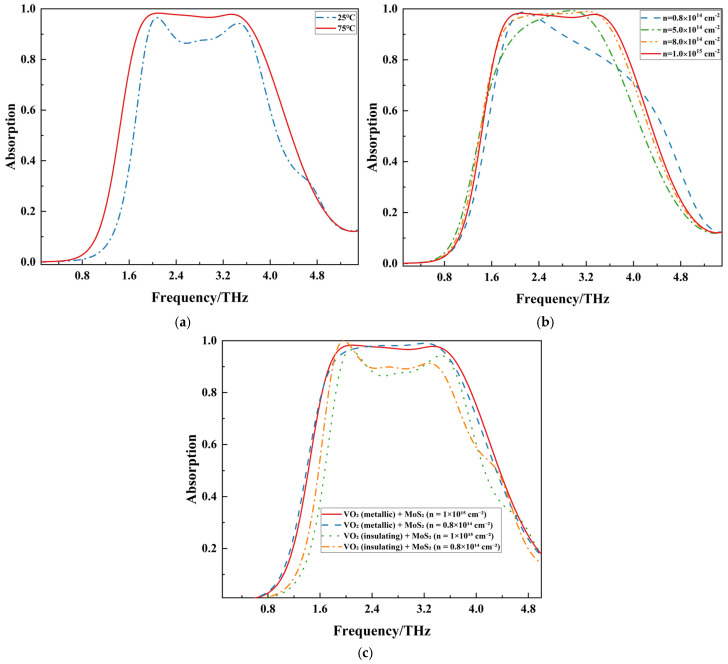
Dual mechanism of dynamic tuning: (**a**) VO_2_ phase transition thermal control (carrier concentration of MoS_2_ is fixed at *n* = 1 × 10^15^ cm^−2^). (**b**) MoS_2_ carrier concentration electrical control (VO_2_ temperature is 75 °C, in metallic state, σ ≈ 9 × 10^4^ S/m). (**c**) Comparison of absorption spectra under four different combinations of VO_2_ and MoS_2_ material states. (States are defined as follows: VO_2_-ON—metallic (75 °C (heating branch, σ ≈ 9 × 10^4^ S/m)); VO_2_-OFF—insulating (25 °C, σ ≈ 30 S/m); MoS_2_-ON—high concentration (1 × 10^15^ cm^−2^); MoS_2_-OFF—low concentration (0.8 × 10^14^ cm^−2^)).

The core competitive advantage of this device lies in its dual dynamic reconfiguration capability driven by both the VO_2_ phase transition and MoS_2_ carrier regulation. Figure 9a,b illustrates how the device maintains optimal performance in a complex electromagnetic environment through the synergy of these two mechanisms.

(1) Thermal-triggered functional switching (macroscopic regulation): As shown in Figure 9a, by adjusting the environmental temperature, VO_2_ undergoes a metal–insulator transition (MIT), and the absorber achieves a macroscopic functional switching from a narrowband double peak to wideband platform. During this process, the carrier concentration of MoS_2_ is fixed at the maximum value of *n* = 1 × 10^15^ cm^−2^ to ensure the optimal impedance matching after the wideband mode is activated.

At 25 °C (insulating state, σ ≈ 30 S/m), VO_2_ behaves as a low-loss medium. At this time, the absorption spectrum is mainly determined by the resonant modes of MoS_2_ and presents a narrowband double-peak feature. As the temperature rises to 75 °C (heating branch, metallic state, σ ≈ 9 × 10^4^ S/m), the conductivity of VO_2_ surges to 9 × 10^4^ S/m, and its plasma frequency *ω_p_* significantly increases, thereby triggering strong localized surface plasmon resonance (LSPR). To ensure the rigor of the results, only stable phase-state parameters are used to guarantee repeatability and physical accuracy. In all simulations of this paper, the heating branch data of VO_2_ were adopted. Specifically, we select the stable fully metallic state (75 °C) as the reference point for analyzing electro-tuning and wideband characteristics to avoid the electromagnetic parameter fluctuations caused by the hysteresis in the critical region. These new modes strongly hybridize and couple with the base mode of MoS_2_, resulting in the splitting of the resonance energy level and smooth transition, ultimately forming a flat absorption platform with a bandwidth of 1.64 THz.

(2) Fine-tuning of electrical triggering performance (precise compensation): Under the condition that VO_2_ remains in a stable metallic state (at a fixed temperature of T = 75 °C), the carrier concentration *n* of MoS_2_ is adjusted through ionic liquid gate technology (Figure 9b). As *n* increases from 0.8 × 10^14^ to 1 × 10^15^ cm^−2^, the surface conductivity of MoS_2_ continuously shifts according to the Drude model, causing the input impedance Z(ω) of the metasurface to approach the free-space impedance Z_0_ at high frequencies (>3.0 THz). This fine-tuning capability compensates for edge impedance mismatch caused by the broadband resonance of VO_2_, enabling the relatively narrowband dual-peak absorption spectrum observed at low carrier concentrations (*n* = 0.8 × 10^14^ cm^−2^) to gradually broaden and merge into a wide, flat high-absorption (*n* = 1 × 10^15^ cm^−2^) spectrum at higher carrier concentrations, achieving an absorption rate exceeding 96.7% across the 1.88–3.52 THz range.

This thermal control + electrical control collaborative strategy has solved the problem of the performance stability of the ultra-wideband absorber in dynamic environments.

To further clarify the individual contributions of VO_2_ and MoS_2_, as well as their synergistic effects on broadband absorption, Figure 9c systematically compares the absorption spectra under four realistic material state combinations. When VO_2_ is in the metallic state and the carrier concentration of MoS_2_ is 1 × 10^15^ cm^−2^ (high-*n* MoS_2_, red curve), the device achieves optimal broadband absorption, with an absorption rate exceeding 96.7% over the 1.88–3.52 THz range, corresponding to a relative bandwidth of approximately 61%.

When the carrier concentration of MoS_2_ is reduced to 0.8 × 10^14^ cm^−2^ (low-*n* MoS_2_, blue curve), the broadband absorption characteristics remain largely preserved. However, a slight reduction in absorption in the low-frequency region (<2.4 THz) results in a minor narrowing of the effective bandwidth, demonstrating the fine adjustment capability of electrical tuning on the spectral profile.

In contrast, once VO_2_ switches to the insulating state (green and orange curves), the broadband absorption platform collapses and transforms into a narrowband resonance centered at approximately 1.95 THz. In this regime, the green curve (high-*n* MoS_2_) and the orange curve (low-*n* MoS_2_) are nearly indistinguishable, indicating that the variation in MoS_2_ carrier concentration contributes negligibly to the overall electromagnetic response when VO_2_ is insulating. The design of this device aims at the stability of ultra-wideband impedance matching. If the adjustment of MoS_2_ causes significant frequency drift, it will disrupt the flat platform in the 1.88–3.52 THz range. The introduction of MoS_2_ is intended to fine-tune the absorption rate to a percentage level (for example, from 92% to 96.7%) after VO_2_ enables wide bandwidth, rather than significantly altering the working bandwidth.

This behavior reveals that the metallic phase of VO_2_ is a prerequisite for establishing broadband impedance matching. During the phase transition, the conductivity of VO_2_ decreases by approximately three orders of magnitude (9 × 10^4^ S/m → 30 S/m), which disrupts the broadband impedance matching condition and results in a localized resonance-dominated response. Furthermore, the electrical tuning sensitivity of MoS_2_ strongly depends on the plasmon-enhanced background provided by metallic VO_2_.

These results clearly demonstrate that the phase transition of VO_2_ plays a decisive role in enabling broadband absorption, while MoS_2_ provides fine electrical modulation only when the broadband channel is activated. The cooperative activation of both mechanisms is therefore essential for achieving ultra-broadband high absorption.

### 3.4. Performance Comparison

To highlight the advantages of this design, Table 2 compares this work with the terahertz absorbers reported in recent years. Although some existing works have achieved dual tuning, they often come at the expense of absorption bandwidth or angle stability. In contrast, thanks to the geometric freedom of the asymmetric multi-nested structure and the collaborative design of dual materials, this device can integrate dual thermal/electrical control capabilities while maintaining ultra-wideband nearly perfect absorption (>96.7%) in simulations. It is expected to alleviate the trade-off between bandwidth expansion and dynamic reconfigurability and can maintain an angular stability of up to 60°. These results suggest that the proposed design strategy may provide an effective route to addressing the bandwidth–function–stability trade-off in terahertz devices.

### 3.5. Fabrication Feasibility

To assess the engineering feasibility of the designed structure, we analyzed the possible preparation processes. The preparation steps are as follows:

Firstly, a 1 μm thick Au layer was deposited on a silicon substrate using electron beam evaporation. Then, a 13.2 μm thick non-damaging SiO_2_ insulating layer was grown using plasma-enhanced chemical vapor deposition (PECVD). Next, a VO_2_ film (about 150 nm) was grown on the SiO_2_ layer through pulsed laser deposition (PLD) or magnetron sputtering. Since the phase transformation property of VO_2_ is relatively sensitive to crystal quality, post-deposition annealing can be performed to optimize the crystallinity of the film. Subsequently, VO_2_ was patterned using electron beam lithography (EBL) combined with reactive ion etching (RIE) to form the required multi-nested C-shaped structure.

A single layer of MoS_2_ should be grown by using chemical vapor deposition (CVD) and can then be transferred onto the patterned VO2 layer using a PMMA-assisted wet transfer technique. Considering the limited thermal stability of MoS_2_, all high-temperature steps should be completed before the transfer of MoS_2_. Using preset metal alignment marks, the MoS_2_ layer was patterned using a mild etching process to align it with the VO_2_ structure.

In addition, for the realization of electrical control, an ionic liquid or ionic gel electrolyte layer can be covered on the top of the structure, and a global gate structure can be constructed by combining a transparent conductive electrode (such as ITO or metal grid) to achieve unified carrier control for the entire metasurface array.

The above process utilized mature micro–nano processing techniques, proving the feasibility of this asymmetric composite structure in experimental manufacturing.

## 4. Conclusions

In this study, we proposed and systematically investigated a MoS_2_/VO_2_ composite terahertz metamaterial absorber based on an asymmetric multi-nested C-shaped structure. Through comparative studies of four different material combination states (VO_2_ metal/insulator × MoS_2_ high/low carrier concentration) and coupled mode theory (CMT) analysis, this work clarified the respective mechanisms and cooperative relationship of the VO_2_ phase transition and MoS_2_ electrical tuning in the formation of broadband absorption. By combining the collaborative design strategy of structural asymmetry and material heterogeneity, this device achieved ultra-wideband absorption (>96.7%, 1.88–3.52 THz, 61% RBW), dual-mechanism dynamic tunability, and wide-angle stability (0–60°). Additionally, this paper discussed global electric control implementation based on ionic gel gate control and the feasibility of device operation under near-ambient conditions, providing support for the practical engineering application of this design. The main conclusions are as follows:

1. Revealed the enhancement mechanism of symmetry breaking: Through geometric asymmetry design to break mirror symmetry, the previously protected dark modes were excited and strongly hybridized with the bright modes. Coupled mode theory (CMT) analysis confirmed that the spectral overlap and coherent interference of the three under-coupled resonant modes (1.60, 2.40, 3.77 THz) enabled the total absorption rate to exceed the single-mode limit (62.3%), maintaining a flat high absorption rate of >96.7% throughout the 1.88–3.52 THz range.

2. Achieved a coordinated dual-tuning mechanism: A new regulatory model of macroscopic functional switch (VO_2_) + precise performance fine-tuning (MoS_2_) was proposed. We verified the complementary effect of the VO_2_ phase transition as a macroscopic function switch and MoS_2_ electrical tuning as a method for performance fine-tuning. The reversible switching of broadband absorption characteristics between a narrowband double peak and wideband platform was achieved. This dual mechanism significantly enhances the flexibility of the device’s dynamic reconfiguration in complex environments and effectively avoids the traditional trade-off issue between wideband expansion and tuning depth.

3. Excellent wide-angle stability: Thanks to the sub-wavelength unit design and local resonance mechanism, this absorber can maintain stable high absorption performance within the wide incident angle range of 0–60° (TE mode) without significant drift in center frequency. This characteristic gives it significant advantages in non-planar object stealth and wide-angle detection applications.

4. Demonstrated the robustness and feasibility of engineering: The device exhibited stable performance over a wide incident angle range of 0–60° (thanks to the sub-wavelength design and the local nature of LSPR), and the tolerance analysis indicated that key parameters (such as gap J1) maintained an absorption rate of >95.9% within a manufacturing error of ±10%, demonstrating excellent process fault tolerance. The single-layer planar configuration simplifies the fabrication process and avoids the alignment challenges of multilayer stacking.

In conclusion, the asymmetric + collaborative design paradigm proposed in this study achieved the integration of broadband absorption and dual-mechanism reconfiguration capabilities in a single-layer structure, providing a reference solution with both physical depth and application potential for the design of high-performance reconfigurable terahertz metamaterials.

## Figures and Tables

**Table 1:** Comparison of resonance mode parameters extracted from CMT fitting for absorber in different VO_2_ phases (MoS_2_: n = 1 × 10^15^ cm^−2^).

VO_2_ State	Mode	Resonance Frequency $\omega_{0}$(THz)	Radiative Loss $\gamma_{r}$ (THz)	Absorptive Loss $\gamma_{a}$ (THz)	Quality Factor Q	Coupling Ratio $\gamma_{r}/\gamma_{a}$
Metallic	1	1.602	0.185	0.333	1.55	0.56
	2	2.402	0.164	0.686	1.41	0.24
	3	3.775	0.380	0.593	1.94	0.64
Insulating	1	1.821	0.217	0.248	1.96	0.88
	2	2.723	0.097	0.124	6.17	0.78
	3	3.739	0.301	0.419	2.59	0.72

**Table 2:** Performance comparison with state-of-the-art tunable terahertz absorbers.

Ref.	Structure Type	Materials	Tuning Mech.	Freq. Range (THz)	Tuning Range/Contrast	Peak Abs.	Max Angle
[30]	Fractal Geometry	MoS_2_/BP + VO_2_	Dual (Therm/Elec)	Multiband	ΔA ≈ 90%	>90.0%	55°
[31]	Double-layer Stack	MoS_2_	Electrical (Single)	Multiband	Independent triband frequency tuning	~99.9%	60°
[32]	Fractal Geometry	Graphene/VO_2_	Dual (Therm/Elec)	3.8–6.23	Dual-broadband ↔ Multi-narrow	~90.0%	55°
[33]	Fractal Geometry	MoS_2_/Graphene	Electrical (Single)	Multiband	Multi-peak Frequency Shift	~99.7%	50°
[34]	Multilayer Metasurface	MoS_2_/VO_2_	Thermal (Single)	0.72–1.92	Broadband amplitude switching	~90.0%	62°
This work	Asymmetric Nested	MoS_2_/VO_2_	Dual (Therm/Elec)	1.88–3.52	Broadband (61%) ↔ Narrowband	>96.7%	60°

## Data Availability

The original contributions presented in the study are included in the article, further inquiries can be directed to the corresponding author.

## References

[1] Cacciari I., Ranfagni A. (2025). Unlocking Terahertz technology with machine learning: A comprehensive review. J. Appl. Phys..

[2] Langde P., Jain T.K., Parate M.R., Singh S.K. (2025). A journey of terahertz communication: An IRS integration perspective. Phys. Commun..

[3] Bi H., You R., Bian X., Li P., Zhao X., You Z. (2024). A magnetic control enrichment technique combined with terahertz metamaterial biosensor for detecting SARS-CoV-2 spike protein. Biosens. Bioelectron..

[4] Wang C., Li X., Wang A., Bai J., Guo Z. (2025). Recent advances in terahertz biochemical sensing technology: Principles, evolution and applications. TrAC Trends Anal. Chem..

[5] Dănilă O., Mănăilă-Maximean D., Bărar A., Loiko V.A. (2021). Non-Layered Gold-Silicon and All-Silicon Frequency-Selective Metasurfaces for Potential Mid-Infrared Sensing Applications. Sensors.

[6] Veselago V.G. (1968). The Electrodynamics of Substances with Simultaneously Negative Values of ε and μ. Sov. Phys. Usp..

[7] Pendry J.B., Holden A.J., Stewart W.J., Youngs I. (1996). Extremely Low Frequency Plasmons in Metallic Mesostructures. Phys. Rev. Lett..

[8] Kang L., Jiang Z.H., Yue T., Werner D.H. (2015). Handedness Dependent Electromagnetically Induced Transparency in Hybrid Chiral Metamaterials. Sci. Rep..

[9] Watts C.M., Liu X., Padilla W.J. (2012). Metamaterial Electromagnetic Wave Absorbers. Adv. Mater..

[10] Han J., Lakhtakia A. (2009). Semiconductor Split-Ring Resonators for Thermally Tunable, Terahertz Metamaterials. J. Mod. Opt..

[11] Serebryannikov A.E., Lakhtakia A., Vandenbosch G.A.E., Ozbay E. (2022). Transmissive Terahertz Metasurfaces with Vanadium Dioxide Split-Rings and Grids for Switchable Asymmetric Polarization Manipulation. Sci. Rep..

[12] Wang D., Xu K.D., Luo S., Cui Y., Zhang L., Liao Z., Cui J. (2023). Dual-Band Terahertz Absorber Based on Square Ring Metamaterial Structure. Opt. Express.

[13] Du X.M., Li T., Yan F.P., Wang W., Zhang L., Bai Z. (2024). Switchable VO_2_ Metamaterial Based on Planar and Vertical Split Ring Resonators for High-Performance Sensing. IEEE Sens. J..

[14] Zhong Z.Q., Wu Q.X., Ling F., Zhang B. (2023). Method for Designing Ultra-Wideband Absorbers Based on Water-Filled Fabry-Perot Cavity with Continuously Varying Cavity Length. Opt. Lett..

[15] Feng H., Zhang Z., Zhang J., Fang D., Wang J., Liu C., Wu T., Wang G., Wang L., Ran L. (2022). Tunable Dual-Broadband Terahertz Absorber with Vanadium Dioxide Metamaterial. Nanomaterials.

[16] Wei C., Deng G., Zhang Y. (2025). Metamaterial-Inspired Ultra-Broadband and Switchable Terahertz Absorber and Polarization Converter Based on Vanadium Dioxide. Phys. Scr..

[17] Xing Y.Z., Chen F. (2026). Tunable terahertz broadband absorber based on single-layer MoS_2_ pattern. Micro Nanostruct..

[18] Liu S., Chen F. (2026). Dual-channel dynamically tunable terahertz broadband perfect absorber based on a VO_2_ and MoS_2_ composite structure. Phys. Chem. Chem. Phys..

[19] Jung H., Jo H., Lee W., Kang M.S., Lee H. (2022). Reconfigurable Molecularization of Terahertz Meta-Atoms. ACS Photonics.

[20] Chen K.L., Wang Z.N., Guan M.Z., Cheng S., Ma H., Yi Z., Li B. (2025). Tunable Ultra-Wideband VO_2_-Graphene Hybrid Metasurface Terahertz Absorption Devices Based on Dual Regulation. Photonics.

[21] Wang X.Y., Chen M., Zhao W.L., Shi X., Han W., Li R., Liu J., Teng C., Deng S., Cheng Y. (2023). Terahertz broadband tunable chiral metamirror based on VO_2_-metal hybrid structure. Opt. Express.

[22] Ye L., Su W., Zou J.F., Ding Z., Luo Y., Li W., Zhou Y., Wu H., Yao H. (2024). Ultra-broadband composite terahertz absorber prediction based on K-nearest neighbor. Opt. Laser Technol..

[23] Liu M.K., Hwang H.Y., Tao H., Strikwerda A.C., Fan K., Keiser G.R., Sternbach A.J., West K.G., Kittiwatanakul S., Lu J. (2012). Terahertz-field-induced insulator-to-metal transition in vanadium dioxide metamaterial. Nature.

[24] Yu N., Capasso F. (2014). Flat optics with designer metasurfaces. Nat. Mater..

[25] Horng J., Chen C.F., Geng B.S., Girit C., Zhang Y., Hao Z., Bechtel H.A., Martin M., Zettl A., Crommie M.F. (2011). Drude Conductivity of Dirac Fermions in Graphene. Phys. Rev. B.

[26] Elliott J.D., Xu Z., Umari P., Jayaswal G., Chen M., Zhang X., Martucci A., Marsili M., Merano M. (2020). Surface Susceptibility and Conductivity of MoS_2_ and WSe_2_ Monolayers: A First-Principles and Ellipsometry Characterization. Phys. Rev. B.

[27] Galarreta C.R., Alexeev A., Bertolotti J., Wright C.D. Phase-Change Metasurfaces for Dynamic Beam Steering and Beam Shaping in the Infrared. Proceedings of the 2018 IEEE International Symposium on Circuits and Systems (ISCAS).

[28] Landy N., Sajuyigbe S., Mock J.J., Smith D.R., Padilla W.J. (2008). Perfect Metamaterial Absorber. Phys. Rev. Lett..

[29] Deng G., Yang J., Yin Z. (2017). Broadband terahertz metamaterial absorber based on tantalum nitride. Appl. Opt..

[30] Zheng S., Huang Q. (2022). Design of a Tunable Monolayer MoS_2_/BP Based Terahertz Absorber with Six Absorption Bands. Opt. Mater..

[31] Ge J., Zhang Y., Dong H., Zhang L. (2022). Independently Tunable Infrared Absorber Using Stacked Molybdenum Disulfide Metasurfaces. Appl. Surf. Sci..

[32] Li J., Liu Y., Chen Y., Chen W., Guo H., Wu Q., Li M. (2023). Tunable Broadband-Narrowband and Dual-Broadband Terahertz Absorber Based on a Hybrid Metamaterial Vanadium Dioxide and Graphene. Micromachines.

[33] Cai F., Kou Z. (2023). A Novel Triple-Band Terahertz Metamaterial Absorber Using a Stacked Structure of MoS_2_ and Graphene. Photonics.

[34] Cui Z., Liu D., Zhu W., Zhang S., Wang L. (2024). A Tunable Terahertz Broadband Absorber Based on Patterned Vanadium Dioxide. Micro Nanostructures.

